# Supplementary material for: One-session treatment for specific phobias: Barriers, facilitators and acceptability as perceived by children & young people, parents, and clinicians
Source: PLoS One. 2022 Sep 15;17(9):e0274424. doi: 10.1371/journal.pone.0274424 (PMC9477316; doi:10.1371/journal.pone.0274424)
Supplement: S1 File — (PDF) [file pone.0274424.s001.pdf]

S1: ***Children and Young People Topic Guide***

Introduction

- Ensure the child has received and read the children's Patient Information Sheet.
- Explain the aim of the chat today.
- Outline what the meeting will be like (drawing/chat); explain that it will take no longer than 30 mins and if they want to stop this will be ok.
- Explain that if the child does not want to answer a particular question then that is OK Explain purpose of digital recording and what happens to the recording you make
- Emphasise that the people who they met for their treatment will not be affected in any way.
- Explain that we will let all children in the study know what we find
- Ensure they are still willing to take part.
- Allow time for any other questions.
- Take child and parent consent/assent.

Phobia (Use drawings where children choose)

- Tell me a little about why you are here. What are you scared of/ what did we see you about?
- How has this affected you? What can you do/not do, how do you feel?
- Who helps you the most? How?
- Is there anyone else you can help/has helped? How?
- What help (if any) have you had before? (parents, friends, other people, health services) in managing your difficulties (mental health services, GP, 3rd sector, self help books). Probe helpfulness/unhelpfulness.

Expectations

- How much did you know/were told before you met [CLINICIAN NAME] ?
- What did you think you were going to do when you came for help/ saw [CLINICIAN NAME] ?
- Was it different to what you thought?
- Did you look forward to coming? Why?
- Could we have made things easier/better for you in any way? How?
- Do you think other children would like to come? Why?

- What type of family/child would like it the most?

Intervention (engagers only)

- What did you do with [CLINICIAN NAME] ? Probe whether it was helpful/unhelpful overall; was it easy/challenging; specific ways it helped/did not help with examples;
- Do you think it has made a difference to you? (Probe: what had an impact; how did you change/not change; was this expected/not expected; the most important change)
- What do you think led to the changes you have just told me about? (Probe: How did the intervention work; was there anything important that happened outside of treatment)
- Do you think the treatment was long enough? Would you like to have had more? How much more?
- What did you think to [CLINICIAN NAME] who supported you (Probe any helpful/unhelpful aspects of their relationship; specific and non-specific factors)

Is there anything that you think would make the intervention better (Probe length of treatment, length of sessions, follow up etc)

Ending

- Thank participant for their time and information
