# Supplementary material for: One-session treatment for specific phobias: Barriers, facilitators and acceptability as perceived by children & young people, parents, and clinicians
Source: PLoS One. 2022 Sep 15;17(9):e0274424. doi: 10.1371/journal.pone.0274424 (PMC9477316; doi:10.1371/journal.pone.0274424)
Supplement: S2 File — (PDF) [file pone.0274424.s002.pdf]

S2: ***Parent and Guardian Topic Guide***

Introduction

- Ensure the participant has received and read the Patient Information Sheet.
- Recap the purpose of the ASPECT research, including ethical review.
- Explain objective of qualitative interview sub study.
- Explain how the interviewee was selected.
- Outline the content of the interview; that it will take no longer than 60 mins (parent) and if they want to stop for a break this will be ok.
- Explain that if the interviewee does not want to answer a particular question then they should not feel under any pressure to do so.
- Remind the interviewee that their answers will be confidential unless they disclose a risk to self or others.
- Explain purpose of digital recording and how the data will be anonymised and stored.
- Emphasise that the mental health services that they receive will not be affected in any way.
- Explain they will be sent a summary of findings when the study is completed.
- Explain the consent process to the interviewee and ensure they are still willing to take part.
- Allow time for any other questions.
- Take consent.

Phobia

- Tell me about your child's phobia and how it has affected their life (*Probe length, and impact*)
- What has been the impact on your own life/ your family's life?
- What help (if any) have you had in managing your difficulties (mental health services, GP, 3rd sector, self help books). *Probe helpfulness/unhelpfulness.*

Expectations

- What were your broad expectations of treatment (*probe not just the interventions we are delivering but more broadly i.e. (a) what treatment they should get from the NHS (b) what outcomes they might expect (help and support, helping them cope, major resolution of symptoms, complete cure*)

Preferences

- When you first agreed to the study did you have a preference for the treatment you wanted to receive (*Probe reasons for preference?*)

- What were your expectations of the treatment you received (*Probe i.e. treatment in its own right, treatment which would help, just something to do; first impressions)*

Engagement

- What motivated you/ stopped you from attending/engaging in treatment?

- Could any of these things have been resolved?

- What (if anything) might stop other children/families attending?

- What type of family/child would this treatment most suit?

Intervention (engagers only)

- What was your experience of the intervention like? (*Probe whether it was helpful/unhelpful overall; was it easy/challenging; specific ways it helped/did not help with examples; was there anything missing)*

- What was your child's experience?

- Do you think that you/your child experienced changes with the intervention? (*Probe: what had an impact; how did you change/not change; was this expected/not expected; the most important change*)

- What do you think led to the changes you experienced/ didn't experience? (*Probe: How did the intervention work; how many sessions, mode of sessions face to face/telephone/mixed; was there anything important that happened outside of treatment*)

- What did you think to the health professional who supported you *(Probe any helpful/unhelpful aspects of their relationship; specific and non-specific factors)*

- What was most helpful (if anything was) – the person or the content of the intervention?

- Is there anything that you think would make the intervention better *(Probe length of treatment, length of sessions, follow up etc)*

Following the intervention (engagers only)

- What did you do following the intervention? *(Probe whether resolved, sought other treatment)*

- How did your experience relate to your initial expectations? (*Probe their decision making about next steps*)

- Would you recommend this treatment to others?

Ending

- Thank participant for their time and information
