# Supplementary material for: One-session treatment for specific phobias: Barriers, facilitators and acceptability as perceived by children & young people, parents, and clinicians
Source: PLoS One. 2022 Sep 15;17(9):e0274424. doi: 10.1371/journal.pone.0274424 (PMC9477316; doi:10.1371/journal.pone.0274424)
Supplement: S3 File — (PDF) [file pone.0274424.s003.pdf]

S3: Clinician Topic Guide

Introduction

- Ensure the Health Professional (HP) has received and read the Information Sheet.
- Recap the purpose of the ASPECT research, including ethical review.
- Explain objective of qualitative interview sub study.
- Explain how the interviewee was selected.
- Outline the content of the interview; that it will not take longer than 60 mins and if they want to stop for a break this will be ok.
- Explain that if the interviewee does not want to answer a particular question then they should not feel under any pressure to do so.
- Remind the interviewee that their answers will be confidential unless they disclose any evidence of malpractice it may be reported to the relevant authority.
- Explain purpose of digital recording and how the data will be anonymised and stored.
- Emphasise that mental health services that they work for will not be directly affected in any way but that the information supplied may help to improve patient services in future.
- Explain that their views would remain confidential and not be passed on to others parties including their employer.
- Explain they will be sent a summary of findings when the study is completed, if requested.
- Explain the consent process to the interviewee and ensure they are still willing to take part.
- Allow time for any other questions.
- Take written consent (or confirm existing receipt of consent if conducting a telephone interview)

Phobia

- What was your understanding of child phobia before beginning the trial? (*Professional*)
- Has the trial increased your awareness of children's phobias? (*Knowledge and understanding of treatment etc)*
- Have you had any experience of treating phobias before? (*Yes / No – If yes in what setting? IAPT training? Public, private, third sector – what approach did you use – CBT or other?)*

Expectations

- What were your general expectations of delivering the OST/CBT treatment?
    - o *Beneficial / Non beneficial intervention*
    - o *Interventions suitable / supportive enough*
    - o *Fit with services/ professional support needs*
- Did you have specific expectations about supervision and training for the trial?
- How did these expectations differ from delivering treatment from other mental health problems/childhood anxiety disorders?

Preferences

- When you first agreed to facilitate the study, did you have the chance to deliver CBT or OST? Did you have a preference for any one of these (*Probe reasons for preference – so why did you prefer this type of intervention? / past experiences with both – positive or negative)*
- When children are allocated to this type of intervention, what if any would be your main reasons for accepting or not accepting them? *(probe type of treatment, workload commitment, service requirements?/ attitudes/ personal preferences)*

Intervention

- What was your opinion of the treatment you delivered (*Probe i.e. treatment in its own right, OST comparison to 'full CBT', first impressions)*
- What did you think of the trial as a whole? (*standard of training, quality, structure, resources*?)
- What was your experience of delivering the intervention like? (*Probe whether it was helpful/unhelpful overall; what did you find positive / negative about it; specific ways it helped/did not help with examples; was there anything missing)*
- Were there any logistical issues/challenges experienced whilst delivering the intervention? (accessibility /*usability of information provided – workbook, work sheets – for you as facilitator and for patient).*
- Personally, how effective do you think this intervention was?
- What contributing factors led to the changes you experienced or didn't experience? (*Probe: all sessions required, mode of sessions face to face/telephone/mixed length of sessions – too long initially or too short?; was there anything important that happened outside of treatment*)

- What did you think to the intervention manual/resources? *(Probe thoughts on the manual – did they refer to it during sessions- what specifically they liked/ disliked during and outside of sessions.*

- Has delivering the (OST) intervention developed your clinical skills? (*Probe – how and in what way)*

- Is there anything that you think would make the intervention better? *(Probe length of treatment, length of sessions, follow up etc)*

- How easy was it to embed/ integrate into your service? (*Probe impact / logistical issues from practitioner level to team and service levels – if known?)*

- Given the choice, would you continue to deliver this treatment?

Ending

- Thank participant for their time and information.
