# Supplementary material for: One-session treatment for specific phobias: Barriers, facilitators and acceptability as perceived by children & young people, parents, and clinicians
Source: PLoS One. 2022 Sep 15;17(9):e0274424. doi: 10.1371/journal.pone.0274424 (PMC9477316; doi:10.1371/journal.pone.0274424)
Supplement: S4 File — (PDF) [file pone.0274424.s004.pdf]

S4: Summary Coding Tables

Table 4: Children & Young People Summary Coding Table

Theme	Codes	Quote
Child's Conceptualisation of Phobia	o Behavioural attribution<br>o Low priority in health hierarchy<br>o Health attribution<br>▪ Rational response to trigger<br>▪ High impact on child wellbeing	***Key Message: Children attributed the origin of their phobia either behaviourally or as a result of an external trigger/precipitating event. They also felt that phobias were not prioritised by others as a high priority in the health hierarchy.***<br><br>*"I had a lot of when I kind of mentioned cos I had to go out school sometimes to go to the counselling things and then and they were a bit like "where are you going?" I'm like "oh yeah I've got a phobia of needles" and they're like "why don't you just have it? it isn't that bad" and then you kind of think, "am I just being dramatic?". You kind of feel a bit bad about it. Yeah, yeah a bit kind of embarrassed I feel like I should be able to have this, why can't I have this? It just doesn't feel too great."*<br>Female, 15 years old, Needle Phobia
Motivation to Seek Help	o Impact of Phobia<br>▪ Child Health<br>● Safety<br>● Physical health<br>● Child distress<br>▪ Family Functioning<br>● Stress on other family members<br>● Family planning burden<br>▪ School Functioning<br>▪ Social Functioning<br>o Previous help seeking/service gaps<br>▪ Awareness of treatment option<br>▪ Treatment availability<br>▪ Professional endorsement and credibility<br>▪ Professional dismissal<br>▪ Professional recognition of phobia	***Key Message: Children were motivated to seek help due to their phobia impacting their health, family functioning, school functioning and social functioning. They also described how they had not sought help previously due to their perception of phobias being at a lower level in the health hierarchy or had received ineffective support previously.***<br><br>*"Well ever since I was little, I've been running into roads to avoid them. And it was, it was getting to the point where it probably needs sorting out, otherwise something dangerous could have happened."*<br>Female, 16 years old, Dog Phobia<br><br>*"No, 'cause I didn't know it was available, so I didn't even know it was a thing. So, I'd never gone to anyone. Even people I'd spoken to about it, I hadn't even realised it was a thing."*<br>Female, 16 years old, Vomit Phobia

Child & Family Coping	o Accommodation<br>o Avoidance<br>o Ignoring/delaying exposure<br>o Minimising<br>o Parents absorbing burden<br>o Parent facilitating exposure not working	***Key Message: Children described accommodation, avoidance, irgorin/delayng exposure as the most common ways they used to manage their phobia. They described how this had impacted relationship with their family and things not working when their parents tried to help them with their phobia.***<br><br>*"Well I couldn't watch certain TV programmes. I couldn't go into some shops. And they even appeared on websites too, which means, so it meant we had to be careful where we looked."*<br>Female, 10 years old, Puppet Phobia<br><br>*"Well, my Mum and Dad tried to help me go under the high roofs of like the bits I was scared of, to try to overcome my fear of it. But it didn't really work."*<br>Male, 10 years old, High Ceilings Phobia
Goals of OST	o Reduction in anxiety<br>o Better understanding of phobia<br>o Better quality of life	***Key Message: Children hoped that OST would help them to feel less anxious about their phobia and reduce the impact it was having on their life.***<br><br>*"I looked forward to it in the sense that I really wanted to overcome the fear because it was getting to a point where it was completely starting to take over my life."*<br>Female, 16 years old, Vomit Phobia
Perceived Efficacy	o Believed it would work<br>o Doubted it would work<br>o Toolkit vs treatment	***Key Message: Children often took a practical approach in their perception of how effective OST would be. They tended to view it as an opportunity to develop skills to reduce the impact it was having on their life.***<br><br>*"I was expecting it to sort of like ease the like impact it had on my life and be able to like give me methods to cope with it rather than just avoidance behaviours, cause that isn't really healthy and it gets in the way of things I want to do."*<br>Female, 16 years old, Vomit Phobia
Process	o Parental presence<br>o Child ownership<br>o Therapist Role<br>▪ Experience and skills<br>▪ Child-centred communication<br>▪ Therapist/child relationship<br>▪ Therapist modelling<br>▪ External independent facilitator<br>▪ Preference for specialist in phobia area<br>▪ Understanding phobia stimuli<br>o Active Participation of child	***Key Message: Children whose parents had been present during the session felt it benefitted them as it helped them to feel more comfortable around new people. They also liked they were able to be in control when designing the session and at each point during the exposure session.***<br><br>*"I mean for yeah for me it was useful kind of having someone there 'cause of course I don't know [therapist name] that well I don't- didn't know the nurse that well so having someone that I know really well as support is something really useful for me anyway."*<br>Female, 15 years old, Needle Phobia

	o Characteristics of intervention<br>▪ Flexible<br>▪ Stepped format<br>▪ Acknowledgement<br>▪ Perceived benefits<br>▪ Pacing<br>▪ Explicit about planning session<br>▪ Long session/intense burden<br>o Efficiency<br>▪ Time<br>▪ Immediate feedback on progress<br>▪ Instantly address misconceptions<br>▪ Minimise need to revisit previous sessions/progress<br>▪ Reduced anxiety build up	*"It was definitely a well thought out, more like, well designed intervention. And the actual like getting, having more control over it, meant it felt more like personalised and you were in control."*<br>Male, 15 years old, Needle Phobia<br><br>***Key Message: Children highlighted the importance of having a therapist who was empathetic and understanding during the process.***<br><br>*"They were understanding, they were patient, they were listening. They were very, very understanding of the situation."*<br>Male, 12 years old, Needle Phobia<br>***Key Message: Children highlighted the efficiency of OST in comparison to CBT.***<br><br>*"If you were to go through the CBT, you'd be going back and forward and then there's petrol and then there's work and then there's school. And all these things that need to be taken into consideration. But, with the one session treatment, it's half a day. You're done."*<br>Female, 16 years old, Vomit Phobia
Child Benefits	o Increased understanding and education<br>▪ Understanding the physical response to emotions<br>o Increased confidence<br>o Reduced anxiety<br>o Improved quality of life<br>o Demonstrable evidence of self-management	***Key Message: Children reported a greater sense of understanding about their phobia and reduced anxiety and this had helped to manage their phobia and improve relationships with others.***<br><br>*"Because it helped me to understand why I was scared of them. It might sound weird but I never really knew why I was scared of them. So I was scared of them, up until now I didn't know why."*<br>Female, 10 years old, Puppet Phobia<br><br>*"You get braver and braver."*<br><br>Male, 7 years old, Dog Phobia<br><br>*"It's just made my life so much less stressful you know, like I don't have to worry about so many things."*<br><br>Female, 15 years old, Vomit Phobia

		"Yeah - I feel a lot happier in myself as a person and around other people. And I think people feel more relaxed around me – like they don't have to constantly be like, like, worrying about what they say or anything."<br><br>Female, 16 years old, Vomit Phobia
Accessibility	o Barriers to treatment<br>o Child readiness<br>▪ Age<br>▪ Developmental understanding<br>▪ Openness to intervention<br>▪ Optimal timing of intervention<br>● Intrinsic (child's own readiness)<br>● Extrinsic (environmental prompts)	***Key Message: Children highlighted the importance of being in a place of acceptance of their phobia to be able to being able access and fully benefit from the treatment.***<br><br>*"I would only recommend it, if the person had kind of accepted their phobia and understood their phobia and felt ready to kind of deal with it, obviously (yeah) cus I feel like with the one session treatment you have to be aware of how you're feeling and you have to not be in denial about how you are, urm and yeah of course if I knew someone that was like that."*<br><br>Female, 15 years old, Vomit Phobia
Suitability	o Complexity of case<br>o Comorbidities<br>o Perceived ability to engage<br>▪ Child's concentration<br>▪ Intensity of long session	***Key Message: Children felt OST it would be important to make sure OST was suitable and complexity of phobia, the presence of comorbidities and additional needs and age would be a factor in this due to the intensity of the session.***<br><br>*"I suppose with dogs you get people who aren't only afraid of dogs but pictures so if it's like that when they can't even see one, that might be a bit harder than like might be easier to take it a lot slowly"*<br><br>Female, 15 years old, Dog Phobia<br><br>*"After like an hour and a half to two hours I got really like cos I've, with me having ADHD and only sitting like I get where he can't sit for ages…and with us sitting there for a long time I get, I kept walking around the room so I just trying to distract myself"*<br><br>Male, 10 years old, Choking Phobia<br><br>*"For some people it might again, the attention span thing cos I'm, I'm quite good at keeping my concentration sometimes urm like for this I can, my concentration will do it. So that wasn't too bad but then for some people again like younger kids might be quite hard to retain it."*

		Female, 15 years old, Needle Phobia
Post-Therapy Reflection	o OST suited to specific phobias<br>o Recommending to others<br>o Expectations vs reality<br>o Toolkit vs treatment<br>o Wider Impact<br>o Recommendations<br>o Ongoing implementation issues<br>o Need for future treatment	***Key Message: Children felt OST would be suitable for children with phobias and would cite their own experience when recommending to others. They felt it was important to manage expectations and view OST as a way to learn tools to manage their phobia as opposed to an overall cure. They described short term and long term effects on the phobia and being able to transfer this knowledge to other anxiety provoking situations. They suggested having a follow-up session would be helpful and a short break during the session to manage the intensity if needed.***<br><br>*"I think probably ones who were like me and really, really ,really scared and then try to avoid things and then come and get a bit better at it."*<br>Female, 7 years old, Dog Phobia<br><br>*"So yeah of course I would recommend it, because it worked for me and I've had so many people try and help me and have nothing work, so if this worked for me I feel like it would work for a lot of other people, because not a lot has worked for me."*<br>Female, 15 years old, Vomit Phobia<br><br>*"I just wouldn't say my fear has gone, my fear of it is still the same but my control over that fear has got better."*<br>Female, 15 years old, Needle Phobia<br><br>*"I still get quite worked up about come quite distressed about my mocks because of my GCSEs (yeah) and they're stressful but I was doing some breathing this morning as well kind of just going up on my way to school cos getting quite worked up and it is it was the kind of the same thing where I went, I was quite nerv, no before my exam, I went through my exam it wasn't as bad as I thought it would be and then I've calmed myself down at that point so it yeah, useful things."*<br>Female, 15 years old, Needle Phobia<br><br>*"Having a follow up if it was about two or three weeks after (yeah) just to kind of go through what I've been through, but not at the same length of time, not for three hours. But, just to kind of go back through it so you've still got it in your brain. Um. But, apart from that, I think everything was really good."*<br><br>Female, 16 years old, Vomit Phobia

Table 5: Parent Summary Coding Table

Theme	Sub-theme	Quote
Parent's Conceptualisation of Phobia	o Behavioural attribution<br>o Health attribution<br>▪ Rational response to trigger<br>▪ High impact on child wellbeing<br>o Low priority in health hierarchy	**Key Message: Parents attributed the origin of their child's phobia behaviourally, as a result of health issues and viewed phobias as low priority in the health hierarchy in comparison to other conditions.**<br><br>*"We genuinely thought it would be something she'd just grow out of and then as it came to a time for her to have vaccinations at school it became very apparent she wasn't growing out of it."*<br>Parent/Guardian, 15 year old girl, Needle Phobia<br>*"That's when the incident with the dog first happened when she was- we were walking past a man with a dog that was very well known for not being a very nice dog and we were walking past. I grabbed hold of my other daughter, [sibling name name], and [sibling name] grabbed hold of [child name]. But the dog just lunged for [child name] and it was a huge Akita-type dog and it went to bite her and just missed her by centimetres and from that day it just escalated. [Child name] fear was just, phobia, not fear, that was when it started."*<br>Parent/Guardian, 12 year old girl, Dog Phobia<br>*"He's had all his vaccinations done and he was very ill as a child, because he got Strep B, when he was born and then he had Quinsy when he was two. So I think that's where the needle phobia came from, to be honest, he's had all his vaccinations, but I think that's due to because him being so young and not so aware. Obviously as he's got older they get more of an opinion and they obviously can't restrain."*<br>Parent/Guardian, 12 year old boy, Needle Phobia<br>*"I was also aware that the NHS don't offer huge amount of support to kids as well, it's not that they don't want to, it's just that there aren't enough resources and I know that, that [child name] anxiety needs would not be huge compared to other kids and so I thought she would be at the bottom of a list and it wouldn't really be a priority so I didn't have huge expectations. "*<br>Parent/Guardian, 8 year old girl, Dog Phobia

Motivation to Seek Help	o Child Health<br>▪ Safety<br>▪ Physical Health<br>● Direct<br>● Indirect<br>▪ Child Distress<br>▪ Sleep<br>o Family Functioning<br>▪ Relationships<br>▪ Stress on other family members<br>▪ Family planning burden<br>o School Functioning<br>o Social Functioning<br>o Previous help seeking/service gaps<br>▪ Parental recognition of phobia<br>▪ Awareness of treatment option<br>▪ Treatment availability<br>▪ Professional endorsement and credibility<br>▪ Professional dismissal	**Key Message: Parents motivation to seek help focused on where the phobia impacted on their child's health, family functioning, school functioning and social functioning. They described the challenges they had faced in accessing timely support.**<br>*"I mean the in the street, if there was insects and it got to the point where I was worried for her safety because, I know she's old enough not to run into the road, but she was almost kind of losing her mind, and it was a worry that she was going to progress to running into the road to get away from insects."*<br>Parent/Guardian, 12 year old girl, Insect Phobia<br><br>*"[Child name] has a needle phobia and it's really affected her because obviously she suffers from a syndrome and therefore needs regular blood tests regarding her kidney function, plus other blood tests that need doing. She also needs- like procedures doing, which obviously is dye injected in and stuff like that, so it's all needles."*<br>Parent/Guardian, 12 year old boy, Needle Phobia<br><br>*"As I said it was just getting worse and worse, it was just getting to a point, it was just so hard it was just getting, every day was an argument just to get out the house."*<br>Parent/Guardian, 7 year old boy, Dog Phobia<br><br>*"She wasn't wanting to go to school on the days they were even discussing the injection. She certainly didn't want to be around when they were having them."*<br>Parent/Guardian, 15 year old girl, Needle Phobia<br><br>*"She didn't want to go to friends' houses or family where there was dogs. So it was starting to really have an impact on, on all of us."*<br>Parent/Guardian, 7 year old girl, Dog Phobia<br><br>*"Everyone knows that CAMHS is there to support mental health, but you don't consider a phobia to be part of the mental health. No-one speaks about phobias, especially when it's things like dogs because they just, people's attitude towards [child name] was just, "Oh, it's just a dog, it's not gonna hurt you. Get on with it" and, I don't know, it's just different."*<br>Parent/Guardian, 12 year old girl, Dog Phobia
Parent & Family Coping	o Accommodation<br>o Ignoring/delaying exposure<br>o Minimising<br>o Parents absorbing burden<br>o Parents responding negatively impacting trust	**Key Message: Parents identified accommodation, ignoring/delaying exposure and minimising as the ways they had coped with managing the phobia. They described the challenges and process of doing this as burdensome and this impacted the relationship and trust with their child.**

		"It became more extreme over recent years, like going, because we walk to school through the park, but then we had to stop doing that because she was screaming absolutely terrified and we had to avoid places like the beach and things like that."<br>Parent/Guardian, 7 year old girl, Dog Phobia<br><br>*"It's distressing 'cause I don't like to see him unhappy and then it creates that worry around- you know- there's still a meningitis jab that he hasn't had. So there's that kind of worry, 'cause I'm sort of, trying to do the best for his health and that's sort of a bit of a contradiction if he's not had that vaccination isn't it? So- and then the dental- the issue around the dental about making life much more complicated. 'Cause he had to have an operation under general anaesthetic which- you know, I don't believe in unnecessary surgery, so that sort of goes against some values but at the same time we have to compromise sometimes because I couldn't see what the other options were really."*<br>Parent/Guardian, 15 year old boy, Needle Phobia
Goals of OST	o Reduction in anxiety<br>o Improved parent management<br>o Improve parent & child relationship	**Key Message: Parents hoped that their child receiving OST would help to reduce their child's anxiety, support them in feeling more equipped to support their child with the phobia and improve their relationship with their child.**<br><br>*"I really wanted her to be able to find ways of managing anxiety, because she was upset by the fact that she's scared of dogs."*<br>Parent/Guardian, 8 year old girl, Dog Phobia<br><br>*"I was just hoping that I'd get some advice on how to deal with [child name]'s phobia."*<br>Parent/Guardian, 12 year old girl, Dog Phobia<br><br>*"Just to be able to do stuff instead of having to plan it and argue about it."*<br>Parent/Guardian, 7 year old boy, Dog Phobia
Perceived Efficacy	o Believed it would work<br>▪ Seen on TV<br>▪ Cure<br>o Doubted it would work<br>▪ Type of phobia<br>▪ Age of child<br>▪ Toolkit vs treatment	***Key Message: There were mixed views around whether they felt OST would be effective. Some parents were sceptical about whether it would meet their child's needs and often adopted a more cautious approach to protect them and their child from disappointment. Parents who believed it would work cited their own understanding of phobia treatment and from what they had seen in television programmes.***<br><br>*"I've seen things like the Speakmans on TV, don't know if you've heard of them? It's on This Morning on TV and they just do very quick sessions with people and just seem to have some really good results."*<br>Parent/Guardian, 10 year old girl, Needle Phobia

		"I thought it would help her, but from the other side I was a bit scared at how can you treat a phobia, especially the one she had, when it so common. And you are just like sort of- you are facing it every single day, because they are- obviously the flies everywhere. How can you treat it with just one session? So I was hoping it would help her, but I just- like I was in doubt (laughs) yeah that we might need some extra help after that."<br>Parent/Guardian, 9 year old girl, Insect Phobia
Process	o Parent Exclusion<br>▪ Good<br>▪ Bad<br>o Parent Involvement<br>o Passive observation<br>▪ Acquisition of new language<br>▪ Acquisition of new tools<br>▪ Understanding and knowledge gained<br>o Child Ownership<br>o Therapist Trust<br>▪ Experience and skills<br>▪ Child-centred communication<br>▪ Therapist/child relationship<br>o Active participation of child<br>o Characteristics of intervention<br>▪ Flexible<br>▪ Stepped Format<br>● Acknowledgement<br>● Perceived Benefits<br>▪ Pacing<br>▪ Explicit about planning session<br>▪ Long session/intense burden<br>▪ Efficiency<br>● Time<br>● Immediate feedback on progress<br>● Instantly address misconceptions<br>● Minimise need to re-visit previous session/progress	**Key Message: Parental involvement in the intervention varied from participant to participant and appeared to be determined largely by clinician preference. Parents that had been present during therapy felt it had helped them to understand more about their child's phobia and how to support them. Parents who were not able to be present also confirmed this view.**<br><br>*"[Child name]'s got separation anxiety disorder, and for us we felt as though the study wasn't handled very well that way, because all of a sudden it went from talking to you guys, sat in a room all together, he knew what was happening, to all of a sudden, then he had to go sit in a room for an hour and half away from us, while we sat in a waiting room. And what we was worried about is, that this anxiety of being separated away from us is now going to mask or totally knock off what he was there for."*<br>Parent/Guardian, 12 year old boy, Needle Phobia<br><br>*"I think it was useful us being there because when [child name] had that initial anxiety about each additional step, I think it was beneficial for her for us to be there to assist with her and just offer those kind of words of comfort which [therapist name] was doing. But knowing that we were there- it was useful having that input in that side of things as well, not only the design of it, but also being there to assist in supporting [child name]."*<br>Parent/Guardian, 7 year old girl, Dog Phobia<br><br>***Key Message: Parents liked the fact that their child was in control during the planning and exposure session. They also appreciated that the therapy took a more active and targeted approach instead of just talking.***<br><br>*"We felt that her wishes were completely respected at all times. And so there was no point during those injections did I feel that [child name] would have one against her will. You know there was never- it was made very clear to [child name] she could say no at any point. I mean obviously once the needle's in then it's too late but you know we understand the practicalities of that but you know up until that point and she could have said no and we felt 100% confident that [therapist name] would have stopped it. And I think that's really important, I think to have that*

	● Reduced anxiety build-up<br>● Reduced social burden/impact	*confidence in somebody, especially with the needle phobia because that element of control is super important I think. So yes it was great, it was really positive."*<br>Parent/Guardian, 15 year old girl, Needle Phobia<br><br>*"It was targeted to the actual problem in a practical way, which really works well with a young person. I think talking just seems, yeah talking it's just I don't know. It's just not as effective as something that's a real, practical, phobia. You know, you can see it, you can smell it, you can touch it, and you have the same sort of treatment. That it's tactile rather than words."*<br>Parent/Guardian, 15 year old girl, Vomit Phobia<br><br>*"When you're setting up the hierarchy, that was really interesting, 'cause that's- I think that's one of the benefits of this type of therapy, is it's generated by the person themselves isn't it? So it is something that is personal to them, which I think is one of the really good things about this."*<br>Parent/Guardian, 15 year old boy, Needle Phobia<br><br>***Key Message: Parents reported that having a skilled therapists who built a positive relationship with their child and adapted their language appropriately was beneficial.***<br><br>*"He worked quite closely with her. It wasn't, sort of, a teacher student relationship over a desk or anything. They were sat together. You know, so it was very much like a team. It was side by side, "We're gonna do this together, come on, let's do what we can do and let's move on." You know, "You've done really well there, great, let's move on to the next step" kind of thing."*<br>Parent/Guardian, 10 year old girl, Needle Phobia<br><br>***Key Message: Parents/guardians reported the single session nature of OST would be beneficial as it would reduce interruption to school and work commitments.***<br><br>*"I think for me, it was the one session treatment. The reason being with like [child name]'s medical issues, we've already missed quite a lot of school for appointments and time-off with illness and you know, various things and so to me like a two hour block out of school, or whatever was better than, I don't know, six or eight half hour."*<br>Parent/Guardian, 7 year old girl, Dog Phobia
Child Benefits	o Increased understanding and education<br>▪ Understanding the physical response to emotions<br>o Increased confidence<br>o Reduced anxiety	***Key Message: Parent/guardians reported following OST children had an increased knowledge and education about their phobia, increased confidence and reduced anxiety.***<br><br>*"I think primarily [therapist name] explaining to [child name] what her reaction was with dogs and how she could understand that and cope with it. I think was really important because that was really helpful for [child name] because she's bright, she's articulate, she's intelligent, she*

		knows, she was able to understand the things that were explained to her by [therapist name], I think that really helped."<br>Parent/Guardian, 7 year old girl, Dog Phobia<br><br>*"I think confidence that [name] knows that, that she can push herself and that some things that cause her huge anxiety, that you don't have to be as anxiety provoking.As soon as she thought they were gonna be- and actually can be strong, she can be brave. I think there's that and that not everything's gonna end up being a disaster, even things that are a bit scary, doesn't actually end up being that scary and what it boils down to it."*<br>Parent/Guardian, 8 year old girl, Dog Phobia
Accessibility	● Barriers to treatment<br>▪ Physical<br>▪ Emotional/psychological<br>● Child readiness<br>▪ Age<br>▪ Developmental understanding<br>▪ Optional timing of intervention<br>● Intrinsic<br>● Extrinsic	***Key Message: Parents/guardians identified physical barriers to access treatment such as lack of transport and needing to take time out of school/work commitments. They also highlighted the meotional barriers and that this type of therapy may be overwhelming. This was particularly prevalent for children with additional needs and comorbidities.***<br><br>*"You'll have to take a bit of time off school, most kids won't mind that. I suppose if- I work part time, so it wasn't so much of an issue really for me, but perhaps some parents, if they work full time, it might be inconvenient to take their kids to some of them. But if it is an actual phobia, I would imagine it impacts on those families lives anyway. You know, if it's significant enough to try and get help for it then, I would imagine you're prepared to invest some time in it as well."*<br>Parent/Guardian, 12 year old girl, Insect Phobia<br><br>*"If they're already anxious about something, why would they want to put their self in that situation? You know, if someone's scared of something, it takes some courage to go and actually face that fear and I think that's what would probably stop most people. If they're not gonna go there, it's for that reason."*<br>Parent/Guardian, 10 year old girl, Needle Phobia<br><br>*"I think it depends on the individual. For anybody with a phobia I think- it depends I suppose, on if the child's got any additional needs or any other problems. They might find it too much in one three-hour period. Might be a bit overwhelming"*<br>Parent/Guardian, 10 year old boy, Blood Phobia

Suitability	o Complexity of case<br>▪ CBT preferred<br>o Comorbidities<br>▪ ASD<br>▪ Other comorbidites<br>o Perceived ability to engage<br>▪ Child concentration<br>▪ Age<br>▪ Learning style<br>▪ Intensity of long session	**Key Message: Parents of children with additional needs and comorbidities discussed their concerns around the suitability of OST for this population and necessary adaptions that should be made.**<br><br>"P: Yes, I hoped he would get the more extended sessions because I know what he's like and a one hit wonder was not going to be enough for him.<br>I: So the Cognitive Behavioural Therapy?<br>P: Yes, yes because he- when we're teaching him anything its repetitive works better for him. Like for instance even maths tables he's been absolutely perfect with his tables, if you leave it for a little while and go back and revisit it a lot of its fallen out and we have to start again. So that seems to be how he learns, by a lot of repetition. So a one hit wonder to me it was a bit disappointing because I thought, well this isn't going stick but that's what I felt sure."<br>Parent/Guardian, 9 year old boy, Insect Phobia<br><br>*"I think you need to get to know the family and the child just a little bit more if you're offering this session of what works for this child and this family. Because as you know Autism is a massive spectrum and what works for one, might not work for the other."*<br>Parent/Guardian, 12 year old boy, Blood Phobia
Parent Benefits	o Perception of child's behaviour<br>▪ Positive<br>▪ Negative<br>o Demonstrable evidence of self-management<br>o Increase own skills in support<br>o Reduced burden of planning events<br>o Enhance child's social inclusion	***Key Message: Parents felt they had experienced benefits in terms of an improved perception of their child's behaviour, increased skills in being able to support them and an improvement in their child's quality of life.***<br><br>*"Because, I think it gives the child and the parent a clearer insight into exactly what's wrong. And that three-hour intensive session made me a lot more aware of how bad it was and what was actually going on."*<br>Parent/Guardian, 10 year old boy, Blood Phobia<br><br>*"Getting her to use the skills she'd learnt and I could tell how thrilled she was that she'd managed to do it. And the relief on her face when she realised she'd had it done and the relief when she realised it wasn't as bad as she'd anticipated. And then when she had the second and then the third she was much, much, more relaxed."*<br>Parent/Guardian, 15 year old girl, Needle Phobia<br><br>*"I feel like I can at least support her now in that and I can say "Well, what if you just wait ten minutes?" she probably won't pass out . Or, you know, if you can, you know, "[child name], what needle would you like?" And give her that voice to say what she would like, 'cause sometimes with the best will in the world, they'll rush in, they don't have the chance to speak to a child. But they do tend to listen to parents. You know, so if you say "Right, just stop a minute, what needle*

		are you using?" You know, "What would you like [child name]?" you know. It might just make that big difference that she feels like she's got the control over it."<br>Parent/Guardian, 10 year old girl, Needle Phobia<br><br>*"We just get out more don't we kid? We go out a lot more, we often walk to school which is nice and, like half term we're gonna be getting out we're gonna do things, go to country park and that. And that's nice to get out and country park and things like that. We still have problems when dogs are off their leads , that's the only time we do have a problem but if they're on leads we just walk by now. But no its totally different, we can actually plan and do things"*<br>Parent/Guardian, 7 year old boy, Dog Phobia
Therapist Role	o Skilled intervention<br>o External independent facilitator<br>o Preference from specialist in phobia area	***Key Message: Parents described the importance of having a skilled and knowledgeable therapist who was external to the situation to deliver the intervention.***<br><br>*"I've tried to do what I can with him, but it's very different when you're emotionally involved. It needs to be somebody- sometimes I think, you've gotta accept that sometimes you need somebody who's outside, who's a professional, who is gonna be able to just walk away from it at the end of the day."*<br>Parent/Guardian, 15 year old boy, Needle Phobia
Understanding Phobia Stimuli	o Understanding Phobia Stimuli	***Key Message: Parents highlighted the importance of having appropriate and realistic stimuli in order to benefit optimally from the therapy.***<br><br>*"She was very good and she kind of knew what things to do help her with it. So, the point, you know, at the end of it, pouring the sick into the toilet, just that horrible noise and even you said that was the worst bit. 'Cause that was very- 'cause that was almost like visually exactly what you'd- and the smell."*<br>Parent/Guardian, 16 year old girl, Vomit Phobia
Post-Therapy Reflection	o OST suited to specific phobias<br>▪ Understanding of phobia type vs severity<br>o Recommending to others<br>o Expectations vs reality<br>o Toolkit vs treatment<br>o Wider Impact<br>▪ Proximal<br>▪ Short term<br>▪ Long Term<br>▪ Distal	***Key Message: Parents felt OST would work better with animal type phobias but views were mixed from blood-injection and injury type phobias. They also highlighted the importance of adaptions to be made for children with additional needs and comorbidities.***<br><br>*"Animal phobias I think definitely. Not sure about other phobias."*<br>Parent/Guardian, 12 year old girl, Dog Phobia<br><br>*"It's not, it's not a definite no. It just needs a little bit more work, that's all. Like I said I think the study would work as it is, would work well for older neurotypical children. I think that the study would work, but for such as children with deeper roots, ASD, additional needs, there's some more planning and preparation needs to be done."*

	o Recommendations<br>o Ongoing implementation issues<br>▪ Individual planning of therapist/commitment<br>▪ Service time/resource time<br>o Need for future treatment	Parent/Guardian, 12 year old boy, Needle Phobia<br><br>***Key Message: The majority of parents who had taken a pragmatic approach in their expectations felt that these had been met.***<br><br>*"Really good. [Child name] doesn't think so because she expected some switch to go off in her brain and she could just go and play with any dog that she saw, but she's adjusting to the fact that it's a gradual process and, you know, she's come that far that she takes it for granted now."*<br>Parent/Guardian, 12 year old girl, Dog Phobia<br><br>***Key Message: Parents reported the short term and long term impact of OST on their child's phobia.***<br><br>*"So, yeah, like I said before, we got super excited and had to go to the pub and have ice cream and cookie in a pub, obviously, where there's dogs there. So, um, 'cause I just like, I was totally amazed about how well she had done. It was just amazing."*<br>Parent/Guardian, 15 year old girl, Dog Phobia<br><br>*"Well certainly sitting in the gardens with a fly on her foot, and she was even talking about it, it wasn't as though she didn't notice it, she did notice it was there, and she was talking about it, she wasn't even wafting it out of the way, she was dealing with it quite well really. So that was quite impressive. And she sat, she did some gardening with me one day, and there was quite a few things around, and one of the plants must have been heavy with bees or something 'cause there was a lot of buzzing coming from it, and she didn't hurtle inside, she probably avoided it a little bit, but she remained in the garden and continued with what she was doing, so that was quite impressive really."*<br>Parent/Guardian, 12 year old girl, Insect Phobia<br><br>***Key Message: Parents made recommendations for how the therapy could be improved. The most prevalent was a brief follow-up session after the therapy had taken place.***<br><br>*"I think maybe I would have liked to- I know that three-hour session was a one treatment session, but maybe another something similar afterwards to see how she's, [child name]'s progressed from where she was then to dealing with a dog again. So, yeah I just feel like there should be a little something just to see how she is with the dog again."*<br>Parent/Guardian, 15 year old girl, Dog Phobia<br><br>*"So, I think maybe after that long session, to show [child's name] that she could be with a dog and do it, then maybe some specific sessions, for example at the beach, so, with [therapist name]*

		at the beach and see how she reacts when a dog runs past. Or, at the park to follow up some more, kind of specific sessions because it was all very controlled and, and it was, it wasn't really like real life."<br>Parent/Guardian, 7 year old girl, Dog Phobia<br><br>***Key Message: Parents highlighted the importance of service organisation during the treatment session and across care pathways in order for treatment to be offered in a timely and effective manner. Some described delays to therapy due to coordination of treatment resources.***<br><br>*"We had like- we had like real problems with getting the session arranged. The lady that we were allocated to actually arrange it, she was- she only worked a couple of days a week and she said like the dog that they had worked on Monday and they didn't have a venue on a Monday that allowed dogs and things. So, it took quite a long time to arrange, well, all of the summer to be honest with you. I think it was, it was the- she was definitely back at school in the September, possibly the October when we actually got the treatment arranged and I did feel that we only actually managed to get it arranged because I contacted [trial manager name]… it just seemed to drag on far too, too long. And then when- when it was arranged it was arranged for a day when I was at work and we only got a week's notice as well, so I couldn't put in a holiday request."*<br>Parent/Guardian, 7 year old girl, Dog Phobia

Table 6: Clinician Summary Coding Table

**Theme**	**Sub themes**	**Quotes**
Therapist Characteristics	o Therapist background<br>▪ Models - therapist preference and experience in particular therapeutic models.	***Key Message: Roles spoken about in context of ASPECT, and how their skills/capacity would fit in with OST. Most clinicians familiar with CBT, but not all had used it to treat phobias.***

	▪ Roles - the therapist role within the service.<br>o Clinical experience of phobia (prior to ASPECT)<br>▪ Theoretical vs Practical - Greater theoretical knowledge over practical experience.<br>▪ Previous experience - previous experience of treating phobia<br>▪ Awareness and understanding of phobia - clinicians knowledge and understanding of phobias including their understanding of risk and Impact on children.<br>● Level of understanding (e.g. good or poor understanding of phobia)<br>● Impact of phobia	*"I think that it could- I think that because the role of a Children's Wellbeing Practitione does work with children that have mild to moderate anxiety or depression, I could see that being able to do One Session Treatment as part of our skills set. Having One Session Treatment there would be beneficial and that would fit in normally within our practices."*<br><br>Psychological Wellbeing Worker - CAMHS
Service Level Perspectives	o Awareness of gap in service provision - they are aware that phobias would not be seen.<br>o Phobias not considered as core business - phobias would not typically be seen by the service.<br>▪ Little demand for phobias (not coming through as referrals)<br>▪ Phobias not seen as a service business?<br>o Prioritisation of other conditions over phobias - phobias are not prioritised for treatment whilst other comorbidities are present, phobias aren't looked for when people are referred in.<br>o Severity of phobia - instances when some phobias are deemed as severe enough to be seen.<br>Phobias seen as part of service<br>o Lack of referrer awareness (e.g. schools, families)	***Key Message: Phobias not seen regularly as they are not prioritised (either by the service or on an individual level, where other presenting issues/goals treated instead), or because of a lack of referrals (ASPECT trial debunked myth that phobias not treated within service).***<br><br>*"So it was usually part of something that was going on so there'd be some family issue and that was, sometimes it wasn't even touched. They would mention it at some point, and it wouldn't even get touched upon. 'Cause the main presenting goal they wanted was something different to, to, to- to look at the relationship with their parents."*<br><br>Senior Counselling Practitioner<br><br>*"It's increased the services awareness and I think the difficulty for our service before, cos we're a specialist CAHMS service, it was the belief that we didn't treat phobias. So erm- but we do actually have a clinical pathway for phobias and we should be treating phobias. So what was great about the trial was that it raised- we have six community CAHMS teams and it raised their awareness that actually we should be accepting referrals for phobias. And yeah so it's been great for our service in that regard that we've probably been accepting more phobias that have come through as a consequence of raising people's awareness of the clinical pathway"*<br><br>Consultant Clinical Psychologist / Core CAMHS Clinical Lead

<table>
<tr><td></td><td></td><td>"It might sound simple when a GP sends a letter to say this young person's got a fear of dogs and the person is reading that referral letter and thinks 'oh it's just a phobia' and they bounce it back. But I think now clinicians are you know- recognise actually we should be taking those cases."

Consultant Clinical Psychologist / Core CAMHS Clinical Lead</td></tr>
<tr><td>Perceived Effectiveness of OST</td><td>o Believed it would work
▪ Coherent link between theory and practice
▪ Knew of good evidence base
o Perceived benefits of OST
▪ Time (less time impact for child)
▪ Momentum/rapport
o Phobia specific efficacy
o Neutral opinion on efficacy
o Concerns
▪ Complexity of case
● Comorbidities - e.g. trauma and autism
o Time
▪ Length of time in distress
● Child distress
● Clinicians managing that distress.
▪ Time concern based on clinician workload
▪ Poor practice effects - unable to practice skills learning between sessions, less able to generalise the learning to the outside world.
▪ Time to build rapport (not enough time to build rapport/develop the therapeutic relationship)
▪ Not enough support
▪ Pressure to cure phobia in one session</td><td>Key Message: Most had positive view of the effectiveness of OST. More appealing to teenagers, less time commitment (clinicians and CYP), easier for CYP to visualise results.

"My excitement was in that kind of adolescent process there is a lot about, 'I want it, I want it now.' Erm are all important and all of those kinds of things and when you mix in that phobia that's stopping some of that. I think if they can see a movement to get rid of it they, the, the kind of motivation for carrying on, if you can push enough forward.I think motivation for carrying on, because they want this contact with their friends, they want to be seen, they you know that kind of thing, egotistical kind of thing … that age range love quick, you know they are into that quick change, 'let's move it and lets move it fast, I'm you know I'm important,' all of those kinds of things I thought it would fit into. So I was excited even at the training stage."

Senior Counselling Practitioner

Key Message: Many concerns. Child lack of trust in process, concerns about building therapeutic relationship (especially in CYP with comorbidities), keeping CYP on task, out of clinicians comfort zone, CYP's gains may not generalise in wider world

"P: So, the idea of being able to turn it around kind of within that one session seemed huge really and sort of an apprehension around sort of knowing as to whether that can happen or not, and yeah, just sort of, nervous about how it would go, kind of, how the young people would respond to the idea that we were proposing they could overcome this in one session.
I: And did you - so you were concerned that the young people wouldn't trust the one session treatment? Is that fair to say?
P: Yeah I suppose to some degree. I wouldn't say that it was a huge concern for me but it was something that I sort of considered when saying I'm apprehensive, you know, how would the young person respond to this idea that you know we're gonna get them in a room and were gonna spend three hours and do this or up to three hours."</td></tr>
</table>

	▪ Children able to focus for this long.<br>o Items on hierarchy may be too ambitious<br>o Concerns about child engagement<br>▪ Child’s view negative<br>● Overwhelming - child view of it as flooding<br>● One shot approach - child worries that this would not be enough<br>● Avoidance<br>● Child’s trust in the process<br>● Concerns about practicalities e.g. sourcing stimulus<br>● Clinician confidence in being able to replicate/produce the intervention.<br>o 3.6 Family view<br>▪ Family investment in intervention<br>o 3.7 Factors affecting intervention choice(n.b. Perceived capability and confidence)<br>▪ Confidence in skills<br>▪ Clinician experience<br>▪ Practical considerations (e.g. more straightforward to set up sessions for CBT)<br>▪ Time<br>● Therapist/service time constraints<br>● Young person time constraints - does it fit their needs?<br>▪ Client choice	Psychological Wellbeing Worker – CAMHS<br><br>*"Erm, its th-, that, I guess that was something I was-, I was maybe a bit unsure about. Because I suppose, when you’re doing the-, I suppose, when you're delivering the low intensity model over a number of weeks, you- you do get like more opportunity to build maybe rapport, build a relationship. Which can, I feel can have a lasting impact on erm you know, maintaining positive change. That kind of thing. So, I suppose in some respects, there was maybe a fear that that-, I think that- well an expectation that, that- that therapeutic alliance might not be strong with OST.”*<br><br>Psychological Wellbeing Worker – CAMHS<br><br>*“Erm, it’s different to the traditional model of the kind of therapy that we provide which tends to be like weekly over a period of time and in-between sessions that you’d expect there to be some kind of homework or more exposure work for the young person to carry out. Because I guess I worried that somebody might be able to overcome their anxiety in the room with me there with them, but then go out of the room and not be able to do that in in the wider world, really, when they got home.”*<br>Psychiatrist<br>*“How much preparation would it take? All those things. I think it was more within our team, it was about the preparation – how do we get the resources where are we doing it, you know? Um How long? Because our usual way of working is to see a lot of children in a week. But from the information and guidance to school where one session treatment you know for that big chunk of time and preparation, it was that kind of – the practicalities of how it would work. And especially, I think, with me and dogs, it was like “How am I gonna get a dog?”, where am I going to run the session, You know. Maybe I guess, because they say ‘Never work with children or animals.’”*<br>School Wellbeing Worker<br><br>**Key Message: Anticipated that it would be more effective for certain phobia types (e.g. animal), than others (e.g. needle)**<br><br>*“I was thinking animal phobias. We could bring something into the actual room. And I’d had – I’ve had conversations with colleagues as well when they’ve kind of got a case or when I was involved in this, we’d have conversations about it and they spoke about their kind of experience with the phobias. And there was maybe animal ones are very specific (yeah, yeah). Use materials in the room.”*<br><br>Interpersonal Therapist

Motivating Factors for Clinicians	o New skill set (n.b. Gaining training more for a new skill set rather than an unmet need)	***Key Message: Opportunity for new skills set, recently qualified clinicians particularly keen.***<br><br>*"I was interested in experiencing it and seeing how that would look, because I've done CBT as normal, that might have been something that I already felt more comfortable with. OST was a new technique and a new type of treatment that I was actually interested in doing it."*<br><br>Interpersonal Therapist<br><br>*"I guess at the time that I did the training for the One Session Treatment, I just, I can't remember exactly at what point it was. But, I was either in the middle of the training year or just finished it. So I was still kind of in the mode of learning new interventions for anxiety. So from that perspective, I was looking forward to having another, I guess, method of being able to do that."*<br><br>Children's Wellbeing Practitioner
OST Training	o Positive<br>▪ Increased understanding of phobia<br>▪ Increased understanding of planning/structuring/ managing session<br>▪ Professional development opportunities<br>▪ Comprehensive<br>▪ Practical<br>o Negative<br>▪ Gap between training and delivery<br>o Recommendations<br>▪ More practical<br>▪ Inclusion of worked examples/case studies<br>▪ Targeted at clinicians dependent on experience/skills/preference<br>▪ Opportunities for supervision and observation prior to delivery<br>▪ Opportunity for top up session	***Key Message: Training should be targeted at clinicians dependant on skills/experience/preference.***<br><br>*"I already felt quite confident with things like CBT (yeah) and I've delivered in the past, so maybe didn't feel like I needed lots of in-depth (yeah) and, erm, training. So it felt right, for me (yeah). Perhaps if I was at the beginning of my training or something like that, then I would've wanted a bit more (yeah)"*<br><br>Psychiatrist<br><br>***Key Message: Training mainly useful in learning how to plan, structure and manage OST.***<br><br>*"Three hours is very different from any other training I think you've had, you know personally that's a long session and I think you've got to have planned, done your hierarchy really knowing what you're gonna do at each stage, and that was - in the training we really got that. So I felt con, I think it could be a little overwhelming thinking of three hours with a client when they come in with their head down and not really prepared, you 'd think 'blinking heck, three hours!?' You know, you need to know, have a real good err kind of hold around what's gonna happen"*<br><br>Senior Counselling Practitioner

	▪ Training on acclimatisation/habituation and anxiety<br>▪ Support around complex cases	***Key Message: Opportunity for top up session would be useful – difficult to retain content from training if large gap between treating first CYP with OST***
Process	o Parent exclusion<br>▪ Parental anxiety<br>▪ Maintaining anxiety/ safety behaviours e.g. the behaviours that parents engage in that may collude with the behaviour.<br>o Parent involvement<br>▪ Modelling<br>▪ Practice (outside of sessions) e.g. continued home practice<br>▪ Education of parent e.g. parent learning about phobia<br>▪ Age dependent (decision to be involved is age dependent)<br>▪ Support in preparation and goal setting<br>▪ Dependent on therapist training background (e.g. purist CBT vs systemic practitioner)<br>o Child ownership/Active participation of child<br>o Therapist role<br>▪ Building therapeutic alliance/ rapport<br>▪ Balancing power dynamics<br>▪ Validating/ normalising client feelings<br>▪ Credible expert<br>o Characteristics of intervention<br>▪ Flexible<br>▪ Stepped format<br>● Acknowledgment<br>● Perceived benefits<br>▪ Pacing<br>▪ Goal setting<br>● Getting the right goal for the hierarchy	***Key Message: Parent involvement can be positive (opportunity to educate them too, parent able to challenge child further), but their role must be clearly defined***<br><br>*"They can explain or clarify or, you know, whereas you wouldn't challenge a child that you didn't know. Whereas the parent can say "Yeah, but remember the time that you…" I don't know "…were near a dog" or whatever. They can come from a place where they know the child, or they can maybe challenge or offer additional information, which is really elpful."*<br><br>School Wellbeing Worker<br><br>***Key Message: Parental involvement can be negative (safety behaviours), it can be useful to complete OST without parent present***<br><br>*"The parent was like wanting to give, like she went in the bag and got like a bottle of water to give to the child. So then I had to say oh sorry can you, because obviously that would interfere with the therapy because it could be seen as safety behaviour."*<br><br>CBT Practitioner<br><br>***Key Message: Clinicians like that it was collaborative process.***<br><br>*"But it was positive because they obviously were a part of it so it was a collaborative process.; So, there was a lot of sort of times spent thinking about or or gauging how that young person wants that step by step to look like, how they want that session to look like and kind of focussing on them having sort of control around that to some degree. So I think that adds to kind of the positivity of it because they're assessing their own goals you know and they're setting, they they've got their own aspirations to what they want to see"*<br><br>Psychological Wellbeing Practitioner<br><br>***Key Message: Validation on recognition during session is important.***

	● Creativity<br>● Planning<br>o Long session/intense burden (session potentially too much for child or not appropriate format)<br>▪ No time for consolidation between sessions.<br>▪ Pressure to get it done<br>▪ Intense session (for both child and clinician)<br>o Efficiency<br>▪ Time<br>● Service<br>● Family<br>▪ Immediate feedback on progress/positive reinforcement/ actively seeing progress<br>▪ Instantly address misconception<br>▪ Minimise need to revisit previous sessions<br>▪ Momentum<br>o Clinician led add-ons (enriching or augmenting or strict changes)<br>▪ Additional clinical time (including long sessions and additional sessions)<br>▪ Client follow up/check in<br>▪ Assessment sessions<br>▪ Homework sheets<br>▪ Supervision systems<br>▪ Sharing evidence of progress/work done in session	*"To give them some validation and say how well they'd done to recognise, you know, that they had a problem and they'd come and sorted it out. And we've come up with a plan and a way which to sort o manage that and then we can reflect back and see, you know, those positive outcomes and actually, you know, for someone at such a young age it's a really valuable lesson or any problems they can come back in the future, with them and I think that's a really, I think that's a really nice message to give children, children of that, of that age really."*<br><br>Psychiatrist<br><br>***Key Message: Clinicians valued the flexibility of OST (able to go at own pace, go backwards, sideways)***<br><br>*"And also being able to you know work when we kind of built sort of a hierarchy or a step by step plan however you wanna kind of term it- the ability kind of in the moment to sort of move up and down that as felt fitting, for a young person. You know so you had the ability to do that so you had that momentum you know whereby you could kind of move forward but equally you can kind of drop back in the moment if you felt that you needed to. Whereas of course in your separate sessions, you can do that but not not in the same way that a one session treatment allows. You know it's a bit more staggered, which I think potentially could sort of stifle kind of progress could be made if you like."*<br><br>Psychological Wellbeing Practitioner<br><br>***Key Message: Intense session for clinician and CYP (CYP will be very anxious and quite distressed), may be useful to have additional therapist present to share load***<br><br>*"I had an assistant psychologist in with me on that one and it were great, because whenever like my attention were dwindling, 'cause it is such a long time to focus, then she'd pick up, we were like a little tag team with* it"<br><br>Clinical Psychologist<br><br>***Key Message: Needle and vomit phobias can be more difficult to treat.***<br><br>*"A little bit attribute of a child as well I suppose, there were no I think you know, I know there has been an examples of needle phobia done in the One-Session Treatment but I think maybe with him it was- yeah just a - I think he just found it incredibly difficult and needed some time in between to process each step he'd made and kind of just reflect on the positive progress he had made."*

		Clinical Psychologist<br><br>*"I think sometimes it can be quite useful for some patients it helps for them to sort of consolidate certain types of learning. Or, so say for example that the person you were seeing was nauseous, the nauseous feeling doesn't just sort of sort of stay with them for just the sort of the session but they'll carry that with them to the- maybe for a couple of hours afterwards. So, that would be for them then to also reflect on the fact that when they come back for the next session they will probably be able to say like well actually, I felt really uncomfortable but actually I wasn't sick, and actually the, the, the saying something along the lines of actually I could find out I could push that symptom more. And, that's good let's give that a go again, whilst within the OST, you don't really have the opportunity to push that symptom in the exact same way because a patient's worked something out the same way."*<br><br>High Intensity CBT Practitioner<br><br>***Key Message: Completing hierarchy in one session ensures no safety behaviours happen in between exposure***<br><br>*"I think it's obviously more straightforward to just do treatment as usual, because it's a similar setup to 8 sessions for treatment of depression would be, and it's just as-usual, isn't it? Um, but clinically, I just don't feel as though, in the context of a phobia and exposure sort of stuff, I just don't feel as though the week's break is helpful. There could be, you know, homework tasks in between sessional work stuff, but it's very hard to know whether or not there were any maintaining behaviours going on, or any safety behaviours. And it's, yeah – so I definitely think that, clinically, it's a better fit to do the single session treatment."*<br><br>Psychological Wellbeing Practitioner<br><br>***Key Message: Some clinicians felt add-ons were needed (e.g. follow up session, child friendly information sheet, ongoing plan created for after session)***<br><br>***Key Message: Photos/videos taken during session may be useful***<br><br>*"I think different things. You know, like um, like a video clip, almost demonstrating um, I don't know. I'm just sitting on the grass, but thinking maybe had we kind of videoed it and given the parent a recording so that, you know, if the child was having difficulties again, going like "This is what we did. This is how we did it" breaking down the stages. I think that second parent who carried on the work I sent her, you know, the stages. And she asked. She proactively asked for kind of the video clips and the photos and, you know, we took pictures and things. And I think she then used those to continue it. Um. But that*

		was that mum being proactive and assertive. So maybe just doing that – putting some kind of pack together "Here's a clip. Here's a picture of your child achieving their goals. These are the stages. These are the things you need to do" You know. Really just breaking it down. 'Cause I sent – with the first mum, who didn't carry it on – I sent her a lot of emails explaining what she needed to do to keep practicing. Yeah. Maybe just that, I would say."<br><br>School Wellbeing Worker
Planning/Logistics	o Location of therapy delivery<br>▪ School based sessions<br>o Time<br>▪ Booking rooms<br>▪ Booking time out of diary - trying to find a period which is 3 hours<br>o Organisation of session tailored to meeting individualised goal<br>▪ Coordinating session with third party availability<br>o Finding Stimulus<br>▪ Time consuming<br>▪ Access to stimulus (sourcing the stimulus)<br>▪ Cost of stimulus<br>o ASPECT research team facilitated support	***Key Message:*** **School based sessions good because they minimise time out of school, but may be difficult for CYP to return to lessons straight after, and better if have clear therapeutic space**<br><br>*"But the bad part of it was that they were in school, y'know being exposed to a phobia, a fear, and then, all be it I spent some time with them afterwards just having real general chats, y'know "what are you gonna do at the weekend?", "what's your next lesson?", erm, y'know trying to kind of just get their anxiety right back down again, and almost distract their mind. They still then had to return to lesson after doing that, with like no contact with their parents or their carer or, no y'know that kind of… Yeah so that, y'know it almost feels like that does need to be done somewhere external. Y'know like you'd go to the doctors for that kind of thing, or you'd go to CAMHS for therapy, y'know. That sort of, because I suppose if it really didn't go right, and you really struggled with it, then that's gonna be another trigger, your school. You're gonna associate school with vomit and then you've got an issue there so. All be it that wasn't the case."*<br><br>School Wellbeing Worker<br><br>***Key Message: Booking room in CAMHS can be difficult, adequate space needed (e.g. when treating dog phobia), booking time out in own diary for 3 hours can be difficult, difficult coordinating evetytyhing, can be struggle to get stimulus (needles etc)***<br><br>*"Other things that I found was, that make it more tricky, was dog phobia. Okay, sort of good to use a variation of dogs, it is, so say for example if you've got- if you're using up to three dogs per three hour ASPECT session it, it worked wonderfully. But, where do you store the dogs? So what, what I'd err was fortunate enough to do was I was working on a Saturday when I did the OST for the dog phobias and I was fortunate enough to use different rooms within the CAMS vicinity. So, so one room would have one dog in another room would have another. Sort of which is how I got round but, in some services that might not necessarily be practical."*

		Children's Wellbeing Practitioner<br><br>***Key Message: ASPECT research team provided support with planning and logistics - implications for if OST delivered outside of a research project***<br><br>*"But for me, even though it was other logistics of finding the venue, the getting the dog and the child and the family. It's more of that. But I felt like the guidance and support that we got was – you know, phenomenal, you know. It was just a phone call any issues we had, you were kind of there and sorting things. So you know, it felt really, really well supported."*<br><br>School Wellbeing Worker
Suitability of Case	o Child readiness<br>▪ Child able to engage with intervention.<br>● Age<br>● Child developmentally able to engage with sessions (n.b future analysis to think about the whether it is more about age or developmental level for suitability - age may need to be taken in the context of developmental level)<br>● Cognitions<br>● Able to sit still<br>● Optimal timing of intervention<br>9.1.1.3.1 Intrinsic readiness<br>9.1.1.3.2 Extrinsic readiness (Child has tangible goal/motivation (e.g. Need to complete task in class)<br><br>▪ Person centred (depends on the individual)<br>o Complexity of case<br>▪ Comorbidities<br>● ASD<br>● ADHD<br>▪ Trauma	***Key Message: May be suited to older children (young children may struggle more with the long session, teenagers keen for quick improvement)***<br><br>*"I think it fits with the adolescence 'I want to move, I want to change this'. Absolutely a time to change it because it's hitting them at you know at a time they want to move away from their parents, move into their friendship groups. A lot of them were sticking to parents because they were so frightened of things (hmm hmm), so couldn't move on, they were kind of trapped in this limbo bit where they wanted to go off with their friends"*<br><br>Senior Counselling Practitioner<br><br>***Key Message: Children's cognitive development should be considered. Additional support (e.g. more psycho-education, more time given to build relationship) could be put in place to help to help CYP that may struggle***<br><br>***Key Message: OST may be preferable where there is time sensitive external reason for treatment***<br><br>*"I think if there was- so say for example if we was against the clock, like the patient was scared of needles but about to have an operation, then in that sort of case then I would sort of strongly suggest they go down an OST type route. If as I mentioned before, in multiple complexities then probably going down the CBT type route when I've got that sort of time, we've got sort of time to explore, got the option to do a lot more between session work to get them to do different types of things too."*<br><br>High Intensity CBT Practitioner

	▪ Other recent / ongoing complexities e.g. Parents recently split up<br>o Phobia specific suitability concerns<br>▪ Severity of phobia<br>▪ Needle phobia<br>▪ Vomit phobia	***Key Message: If comorbidities are present, they can make a case more complex (ASD, ADHD, other mental health issues such as anxiety or trauma, adverse childhood experience, or being 'looked after'/in care). Rigid thinking in those with ASD may push CYP further, but could be more difficult to shift thinking patterns. Some services do not treat these more complex phobia cases***<br><br>*"I think y'know like children with autism, and I know that- I suppose, thinking about children who've previously experienced an adverse childhood experience, or a childhood trauma, looked after children... Y'know, it's how does that impact on the feelings that they're already trying to kind of cope with, manage, process if you like. I know that anxiety is quite prevalent in children with autism, so that's already there in life, if you like. And then there's that more specific anxiety and fear around the phobia. And it almost feels like that's an extra layer for them? And likewise with children who've experienced trauma, they're in that, y'know that hypervigilant mode all the time. And it's hard and really, y'know, really overwhelming for them. And then you're kind of adding that extra layer on top of that"*<br><br>School Wellbeing Worker<br><br>*"P: Yeah. I wonder whether – you know, if there was comorbid with a young person who'd got autism, it might be more effective for them, I don't know.*<br><br>*I: Yeah? Why do you think that could be?*<br><br>*P: Um. I suppose because maybe if their thinking "I've got to achieve this" because of their rigid thoughts about you know, achieving this by the end of the session that they might really push themselves to work through it (yeah, definitely). There's a time to achieve it by."*<br><br>Interpersonal Therapist<br><br>*"If we're just thinking about specific ASD, ones, and for me with ASD, I think for me it was just considering how difficult it is for them to, y'know sometimes manage transitions from one thing into the next, and they need that preparation beforehand. Sometimes they like to know what's happening next. Well obviously we should be explaining that, absolutely, in the One Session Treatment. But I think that perhaps maybe the speed they move from one activity to the next might influence the treatment, I don't know"*<br><br>CBT Practitioner/ Safe Care Lead

		"As a service, we were actually – we were fairly brutal, to be honest. Um, we would carefully screen referrals and we would um, we would reject referrals prior to assessment sometimes. So, we would take a look at the presenting problems. So, for example, if the – if it straight up said that somebody had received a diagnosis of bipolar disorder in the past, we would say "Okay, we're a low intensity service offering mild to moderate stuff" we would signpost as appropriate, because it's a heartache for them, really, coming in and feeling heard and then being sent away."<br><br>Wellbeing Practitioner<br><br>*"Because as I said the severity of it and that came about as a result of her mother's ill health and time in hospitals so you know when I said earlier about potentially there's sort of, not always, what I'd constitute as trauma but potentially some form of you know you know experience that you know, significant experience that they've had that then so that one is one of those where potentially had they not got where we got, you know, she might've needed further work it may be that she would require further work at a later stage you know when you follow-up such as that I don't know"*<br><br>Psychological Wellbeing Worker – CAMHS<br><br>***Key Message: Distress levels should be considered***<br><br>*"I: ...When children are allocated to this type of intervention what if any would be your main reasons for accepting or not accepting them onto the intervention?*<br><br>*P: I think it depends err, on, level of distress it would be, so say if the client came into the sort of the clinic room, highly distressed about the idea of within the certain amount of time that they're gonna be doing X, Y and Z, (yeah) that may not be sort of feasible to do something like that,"*<br><br>High Intensity CBT Practitioner<br><br>***Key Message: Phobia severity should be considered. Anything beyond mild-moderate phobia may not be suitable***<br><br>*"Because there was another young person I saw that, erm, actually didn't even progress beyond the assessment appointment. It was kind of really severe, an impairing phobia, but it was never gonna be realistic for that to have been cured in one session. But actually we could perhaps have set a, you know, a different goal that would've meant that the phobia was still around but more manageable."*

		Psychiatrist<br><br>***Key Message: Needle phobias may not be suited to OST – difficult to treat in 3 hours due to safety monitoring, may be more useful to build up to having injection after spending several sessions building it up to it and visiting alternative settings***<br><br>*"Unfortunately, I don't think it was very successful. On reflection, I think it was quite specific to needles. I do think the three hours – it didn't work for either of them in the sense that they weren't able to have the vaccinations. No, sorry, one of them wanted a vaccination, the other one just wanted to learn about them, I think. I know one – that was it, he wanted to watch someone have an injection done. … I had contact with a vaccination nurse who was kind of asking about the treatment and I'd explained, you know, that they'd had the treatment -and they were kind of sharing their concern that they didn't think that the one session treatment worked cause they've had other young people and their personal thing was that actually when people have had CBT sessions that, you know, multiple sessions, that was much more effective in their experience of needle phobias"*<br><br>Interpersonal Therapist<br><br>***Key Message: Main point: Vomit phobias may not be suited to OST – fear of becoming sick in days following, not able to expose CYP to actual feeling of nausea***<br><br>*"But she also had- so- and again when we're talking about that core beliefs, there was- there was the vomit phobia which was bad for her, but her- her core belief was that it was gonna make her sick. So she was still really anxious after the OST 'cause she was waiting to get sick. So it was about how do you target that in a fear hierarch in an OST? 'Cause it- it- it was, you know, she didn't have any rules about when it would happen, it was just she didn't sleep for like a night and a day or something because she was so worried that she was gonna get sick from it."*<br><br>Psychological Wellbeing Worker – CAMHS<br><br>***Key Message: Suitability criteria would be useful***<br><br>*"I think it's having a sort of suitability criteria. That would be, more beneficial for the OST, but also kind of like what I was saying before, if there is complexities having that, that almost wiggle room to say actually, actually, we, we can do, more. We can."*

		High Intensity CBT Practitioner
Post Therapy Reflection	o Effectiveness of OST<br>▪ Parent and Child Outcomes<br>● Generalised outside of the phobia<br>● Extent of impact on families life<br>● Reduced anxiety<br>● Improved confidence<br>▪ Phobia specific<br>▪ Progression vs Completion<br>● Improved management vs Cure<br>● OST as primer for CBT/further treatment<br>▪ Success factors<br>● Clinician Factors<br>Therapist confidence<br>Therapist knowledge and engagement with stimulus<br>Facilitating child engagement/Therapeutic relationship<br>Clinician modelling<br>Clinician lived experience<br>▪ Family Factors<br>● Parental involvement<br>Parental investment<br>Parents stepping back<br>Parent explanation and information<br>● Child motivation<br>● Practice following the sessions<br>▪ Intervention factors<br>● Immediate positive reinforcement<br>● Ambitious hierarchy<br>● Evenly spaced hierarchy (even feeling gaps between things)	***Key Message: Positive outcomes – improvements seen in other areas, improved confidence (during the session and after), OST could be used as primer for further CBT, didn't always completely cure the phobia***<br><br>*I think y'know with, particularly one of the ones that I did in a CBT, she did absolutely outstandingly, and erm her mum was then able to use the principles that we'd used in her sessions to support her with her phobia of spiders as well. So that went, as well as the vomit phobia! And she could use the general way of thinking, and challenging thoughts, in her early teenage life! So I think mum was particularly pleased with that, and it made quite a big difference*<br><br>School Wellbeing Worker<br><br>*"So although the three hour session was good at getting it, it, it got err, a lot of erm, it got him to a point where he was able to do a lot more, the condition wasn't anywhere near treated"*<br><br>High Intensity CBT Practitioner<br><br>***Key Message: Clinician based success factors - Clinician must be fully confident in OST, knowledgeable about stimulus, model good behaviour, establish good relationship, OTT praise, step back at right time***<br><br>*"I think you just need to be super prepared beforehand and have everything there, everything ready and a real confidence in the approach as well, I think if you doubt it as a clinician that's gonna really come across to the young person or the parent and - children are you know, they've got these little antenna's that they do pick up on any any doubt in you as a therapist. "*<br><br>Consultant Clinical Psychologist / Core CAMHS Clinical Lead<br><br>***Key Message: Parental presence can be success factor, but they must know when to step back. Child's own motivation is key to success.***<br><br>*"I think having her Mum engaged in the session but with clear rules, not to step in, as potentially Mum could've sabotaged by, and the girl even commented in in when we were looking at some of the videos at, 'Mum you're just as bad as me' and again we had a bit of a laugh about that and how you know, maybe that's one of the maintaining factors. Maybe if it's difficult for your Mum to do then maybe your Mum could join in the treatment, we'll get two for the price of one erm and and yeah so that was having clear erm rules of 'I'm not gonna make you do anything you don't want to do, I'm not gonna force you' but then also some clear rules for Mum if - she wanted her Mum to be in the room but really the clear rules of Mum to be there as her support but not to step in and erm and really talking to them quite a lot about avoidance and about how there'd be real*

	● Flexibility /contingency<br>● Appropriate resource/Realistic stimulus (looking real)<br>● 10.1.4.3.6 Clear Goals<br>▪ 10.1.5 Limiting factors/CBT better adapted<br>● Working through hierarchy too slowly<br>● Difficulties establishing rapport<br>● Lack of child engagement/motivation<br>● Clinician lived experience<br>● Pressure to get the children to the end<br>▪ Expectations vs reality<br>● More time needed to complete OST<br>● Disappointed when they don't complete<br>● Child got further than expected/had a bigger impact than expected<br>● Clinician enjoyed it more than expected<br>● Child coped with length of the session<br>● Children engaged well and took the lead<br>● OST was quicker than initially thought<br>● Session length wasn't too long<br>o Embedding OST within clinical services (n.b. Consider implementation issues of the study when writing this up)<br>▪ Recommendation for streamlined approach to deliver in specified pathway vs ad-hoc approach adopted by various therapists across service.<br>▪ Possible to embed it into services<br>▪ Barriers to service take up<br>o Clinician benefits<br>▪ Learning experience<br>● Increased confidence working with exposure and anxiety (more about generalisation)<br>● Training	*temptation during the exposure to avoid and to not look at the videos or the pictures and der der der der, to really be clear with them about what what the expectations were during the exposure part."*<br><br>Consultant Clinical Psychologist / Core CAMHS Clinical Lead<br><br>***Key Message: OST based success factors - allows for quick progress and lots of immediate positive reinforcement, setting an ambitious but evenly spaced hierarchy leads to more success, OST allows for flexibility, momentum built quickly and enables fast progress***<br><br>*"I think the hierarchy was a large part of the success. So um, so it goes sort of 10, 25, 25, 30, 45, 45, 50, 60, 70, 80, 85. So they were quite evenly spaced in as much as there wasn't like a 90 and then 100. Or a 70 and then a 90. There were lots of things on the hierarchy and opportunities to bridge the gap between certain things."*<br><br>Wellbeing Practitioner<br><br>***Key Message: High quality, realistic stimulus contributed to success***<br><br>*"Exactly, exactly (laughs). I think like if, you know, if I suppose a comparison would be with a dog phobia. As much as you could get a child playing with, I don't know, stuffed teddy dogs, erm, you just, you wouldn't have the confidence that they could necessarily play with a real dog."*<br><br>Psychiatrist<br><br>***Key Message: Limitations of OST - Difficulty in being able to build a relationship with CYP remained a concern for some therapists after OST delivery, potential for negative effect on child in they don't complete***<br><br>*"What do- what implications do you think for the child would feeling like they had failed at their maybe their treatment or their phobia mean?*<br><br>*P I guess there's feeling like going away and not- not feeling like they wanted to continue the progress maybe? Not kind of feeling invested in it, you know just thinking "well I can't change this" so-*<br><br>*I Yeah*<br><br>*P Maybe a bit of hopelessness? Whereas I suppose if you've kind of developed a lot of the skills around you know kind of managing anxiety and noticing and that kind of thing I think it's a skill set then that they could use and take away."*

	● Clinical skills<br>● Increased understanding of phobia<br>▪ Enjoyed taking part<br>▪ Opportunity for innovation<br>▪ Positive observable outcome<br>o Post ASPECT Treatment preference<br>▪ CBT<br>▪ OST<br>o Recommendations for OST structure<br>▪ Follow up session<br>● Relapse prevention<br>● Flexibility to do more<br>▪ More time for psychoeducation/scaffolding<br>▪ Dedicated space in sessions to focus on coping with anxiety during exposure<br>▪ Suitable for PWPs<br>▪ More sessions to build rapport<br>▪ Flexible timing<br>▪ Splitting into two longer sessions (vomit phobia having an additional session at a later date)<br>o Recommendations for treatment delivery (potentially going to be dependent on clinician background)<br>▪ Resources<br>● Enough time for planning and stimulus acquisition<br>● Child friendly resources<br>● Central hub with recommended resources<br>▪ Homework leaflets<br>▪ Outside of school setting<br>▪ Developing a suitability criteria<br>▪ Having another professional in the room for support<br>o Change in perspective (how aspect changed knowledge of experience of services - in the context of their own services)	Clinical Psychologist<br><br>***Key Message: OST does have a place in some services - some felt it was good opportunity for cross pathway working, reduced administrative burden on service (due to less sessions). In response to ASPECT trial, treatment team set up in one service to see phobias***<br><br>*"And actually the benefit that that then had for those practitioners who've then seen young people with say a simple phobia and they've got better, it's boosted their motivation. So I think what it's, what it's now given us is something that we can go back to the managers and say actually this is you know, on lots of different levels, there's a real incentive for actually having some presentation be seen much quicker even if there are only mild to moderate, it actually has a benefit for those clinicians in terms of their motivation and feeling like skilled therapists"*<br><br>*Consultant Clinical Psychologist*<br><br>*"It's increased the services awareness and I think the difficulty for our service before, cos we're a specialist CAHMS service, e is was the belief that we didn't treat phobias. But we do actually have a clinical pathway for phobias and we should be treating phobias, so what was great about the trial was that it raised. We have six community CAHMS teams and it raised their awareness that actually we should be accepting referrals for phobias. Yeah so it's been great for our service in that regard that we've probably been accepting more phobias that have come through as a consequence of raising people's awareness of the clinical pathway."*<br><br>*Consultant Clinical Psychologist*<br><br>***Key Message: Barriers to offering OST in future include that it is challenging fitting in phobia work to an already stretched service***<br><br>*"My worry is that we're just so stretched that, even though it- it's a good kind of quick intervention to deliver if we're not offering a service for phobias at the moment I would wonder kind of how we would- how that would be pitched. Because that's- it's still offering an extra service that we don't at the moment. Even though it's something that could be quite limited, and actually, as I've described, it could have wider ranging effects and impacts on the young person's mental health, which might mean they need less of other interventions."*<br><br>*Psychiatrist*

	o Comparison to CBT	*"I think it would fit within services but I think the problem is some elements of the practicalities of it. So, say for example, I had, err, one patient who cancelled two OSTs sort of sessions. So that's almost, that's the equivalent of, if they cancelled one CBT session that's the equivalent of one hour, but with the OST that was almost the equivalent of seven eight hour. So particularly, that, that was with having to get the resources, having to have the, I think in this case it was sort of having to locate and get a fake arm, because I was doing needlestick phobia, getting another member of staff who was a phlebotomist come in the session with me, so it was all these types of things that would err, in some ways it would make it a bit more tricky."*<br><br>*High Intensity CBT Practitioner*<br><br>***Key Message:*** **Increased therapists confidence and skills, was a rewarding experience**<br><br>***Key Message:*** **Mixed opinions on whether they would continue to deliver OST – some very enthusiastic about continuing to offer OST, one therapist did not want to due to lack of relationship building**<br><br>*"I think I would continue to deliver the CBT one, but I don't think I would continue with the one session one as it is now. If it was, if it was, if it was more relationship led if you like. And person centred in that way, then I would probably, y'know be happier to do it. But that was the missing part for me, and I think that really, makes a huge difference in any therapeutic, y'know piece of work and relationship."*<br><br>School Wellbeing Practitioner<br><br>***Key Message:*** **Some recommendations affect definition of OST. Key recommendations for OST include adding a follow up session, more time to focus on psychoeducation, sessions run by career mental health professionals as more likely to treat mild-moderate phobias**<br><br>*"I think we would find out how things have gone and if things have maybe sort of regressed a bit. Then encourage them to think about what they need to do in order to build up again and overcome things. So I'd use it as a, as a way of being able to recap on the learning that they did in the OST and the previous, in the appointment before that."*<br><br>Children's Wellbeing Practitioner
