# Supplementary material for: One-session treatment for specific phobias: Barriers, facilitators and acceptability as perceived by children & young people, parents, and clinicians
Source: PLoS One. 2022 Sep 15;17(9):e0274424. doi: 10.1371/journal.pone.0274424 (PMC9477316; doi:10.1371/journal.pone.0274424)
Supplement: Attachment — Submitted filename: Response to Reviewers.docx [file pone.0274424.s006.docx]

**Response to Reviewers**

| **Editor Comment** | **Author Response** |
| --- | --- |
| Please ensure that your manuscript meets PLOS ONE's style requirements, including those for file naming. The PLOS ONE style templates can be found at  https://journals.plos.org/plosone/s/file?id=wjVg/PLOSOne_formatting_sample_main_body.pdf and  https://journals.plos.org/plosone/s/file?id=ba62/PLOSOne_formatting_sample_title_authors_affiliations.pdf | The revised manuscript and file names have been updated in line with PLOS ONE submission style templates. |
| Please update your submission to use the PLOS LaTeX template. The template and more information on our requirements for LaTeX submissions can be found at http://journals.plos.org/plosone/s/latex. | We contacted the PLOS LaTex helpdesk for technical support. In our correspondence with Glenn Jackson he advised that our submission would not need to be updated as the manuscript has been built using Microsoft Word and to submit in a docx format. Therefore, we have uploaded our revised manuscript (tracked changes and clean version) in docx format. |
| Thank you for stating the following financial disclosure: "This research was funded by the National Institute for Health Research under its Health Technology Assessment Programme (Grant Reference Number: HTA 15/38/04). The views expressed are those of the author(s) and not necessarily those of the NIHR or the Department of Health and Social Care."  Please state what role the funders took in the study. If the funders had no role, please state: "The funders had no role in study design, data collection and analysis, decision to publish, or preparation of the manuscript.  If this statement is not correct you must amend it as needed.  Please include this amended Role of Funder statement in your cover letter; we will change the online submission form on your behalf. | We confirm that the statement “he funders had no role in study design, data collection and analysis, decision to publish, or preparation of the manuscript.” This has been added to our covering letter. |
| In your Data Availability statement, you have not specified where the minimal data set underlying the results described in your manuscript can be found. PLOS defines a study's minimal data set as the underlying data used to reach the conclusions drawn in the manuscript and any additional data required to replicate the reported study findings in their entirety. All PLOS journals require that the minimal data set be made fully available. For more information about our data policy, please see http://journals.plos.org/plosone/s/data-availability.  Upon re-submitting your revised manuscript, please upload your study’s minimal underlying data set as either Supporting Information files or to a stable, public repository and include the relevant URLs, DOIs, or accession numbers within your revised cover letter. For a list of acceptable repositories, please see http://journals.plos.org/plosone/s/data-availability#loc-recommended-repositories. Any potentially identifying patient information must be fully anonymized.  Important: If there are ethical or legal restrictions to sharing your data publicly, please explain these restrictions in detail. Please see our guidelines for more information on what we consider unacceptable restrictions to publicly sharing data: http://journals.plos.org/plosone/s/data-availability#loc-unacceptable-data-access-restrictions. Note that it is not acceptable for the authors to be the sole named individuals responsible for ensuring data access.  We will update your Data Availability statement to reflect the information you provide in your cover letter. | While the data presented does not contain any direct identifiers, due to the sensitive nature of the data presented and vulnerable participant group, we would not be able to make the dataset publicly available due to ethical reasons. The following wording can be added to the Data Availability statement: “Any requests for sharing of data can be made to the corresponding author or Sheffield Clinical Trials Unit (ctru@sheffield.ac.uk**)**. The ASPECT trial management team will consider the sharing of data on a case-by case basis in line with the ethical approval and patient information sheets.” This information has been added to our covering letter. |
| Please note that in order to use the direct billing option the corresponding author must be affiliated with the chosen institute. Please either amend your manuscript to change the affiliation or corresponding author, or email us at plosone@plos.org with a request to remove this option. | The corresponding author’s affiliation has been updated to Leeds & York Partnership NHS Foundation Trust. We have been asked by the organisation for any invoices for payment to be addressed to : FAO Sarah Cooper, Leeds & York Partnership NHS Foundation Trust, RGD Payables 4425, Phoenix House, Topcliffe Lane, Tingley, Wakefield, WF3 1WE. |
| Please review your reference list to ensure that it is complete and correct. If you have cited papers that have been retracted, please include the rationale for doing so in the manuscript text, or remove these references and replace them with relevant current references. Any changes to the reference list should be mentioned in the rebuttal letter that accompanies your revised manuscript. If you need to cite a retracted article, indicate the article’s retracted status in the References list and also include a citation and full reference for the retraction notice. | We have reviewed our reference list and included a list of changes in our response to reviewers letter. |
| Please amend your Response to Reviewers letter to include a point by point response to each of the points made by the Editor and / or Reviewers. Please follow this link for more information: [http://blogs.PLOS.org/everyone/2011/05/10/how-to-submit-your-revised-manuscript/](http://blogs.plos.org/everyone/2011/05/10/how-to-submit-your-revised-manuscript/) | We have amended the letter to include a table with point by point responses to editors and the reviewer. |
| Thank you for stating the following financial disclosure: "This research was funded by the National Institute for Health Research under its Health Technology Assessment Programme (Grant Reference Number: HTA 15/38/04). The views expressed are those of the author(s) and not necessarily those of the NIHR or the Department of Health and Social Care."  Please state what role the funders took in the study. If the funders had no role, please state: "The funders had no role in study design, data collection and analysis, decision to publish, or preparation of the manuscript.  If this statement is not correct you must amend it as needed.  Please include this amended Role of Funder statement in your cover letter or in the Author Comments section; we will change the online submission form on your behalf. | We confirm that the statement “the funders had no role in study design, data collection and analysis, decision to publish, or preparation of the manuscript.” This has been added to our covering letter. |
| Please note that in order to use the direct billing option the corresponding author must be affiliated with the chosen institute. Please either amend your manuscript to change the affiliation or corresponding author, or email us at plosone@plos.org with a request to remove this option.  Please provide your response in the "Author Comments" section. | The corresponding author’s affiliation has been updated to Leeds & York Partnership NHS Foundation Trust. We have been asked by the organisation for any invoices for payment to be addressed to : FAO Sarah Cooper, Leeds & York Partnership NHS Foundation Trust, RGD Payables 4425, Phoenix House, Topcliffe Lane, Tingley, Wakefield, WF3 1WE.  Our response has been provided in the Author Comments section. |
| Can you please upload an additional copy of your revised manuscript that does not contain any tracked changes or highlighting as your main article file. This will be used in the production process if your manuscript is accepted. Please amend the file type for the file showing your changes to Revised Manuscript w/tracked changes. Please follow this link for more information: [http://blogs.PLOS.org/everyone/2011/05/10/how-to-submit-your-revised-manuscript/](http://blogs.plos.org/everyone/2011/05/10/how-to-submit-your-revised-manuscript/) | The clean version of our manuscript has been updated with all tracked changes accepted. |
| We note your current Data Availability Statement says: "While the data presented does not contain any direct identifiers, due to the sensitive nature of the data presented and vulnerable participant group, we would not be able to make the dataset publicly available due to ethical reasons. Any requests for sharing of data can be made to the corresponding author or Sheffield Clinical Trials Unit. The ASPECT trial management team will consider the sharing of data on a case-by case basis in line with the ethical approval and patient information sheets."  Before we can proceed, please provide the contact information for the Sheffield Clinical Trials Unit. We will update your Data Availability Statement on your behalf once you provide the necessary information. | While the data presented does not contain any direct identifiers, due to the sensitive nature of the data presented and vulnerable participant group, we would not be able to make the dataset publicly available due to ethical reasons. The following wording can be added to the Data Availability statement: “Any requests for sharing of data can be made to the corresponding author or Sheffield Clinical Trials Unit (**ctru@sheffield.ac.uk)**. The ASPECT trial management team will consider the sharing of data on a case-by case basis in line with the ethical approval and patient information sheets.” |
| **Reviewer Comment** | **Author Response** |
| This article reports on a qualitative sub-study as part of the ASPECT trial, to assess acceptability of one session treatment for specific phobias and common barriers and facilitators, as identified through interviews with children/young people, parents and clinicians who deliver treatment. The article clearly presents the key themes with illustrative quotations and provides sufficient detail regarding the methodology. | Thank you! |
| The authors do not provide access to all data underlying findings. It would be helpful to provide a more detailed data availability statement regarding restrictions on availability of data in line with the data availability policy. If there is no opportunity to access data other than the quotations selected for the article and the summary coding table, the statement could usefully state this and comment on the selection of data presented in this article and supplementary material. I feel that significant data are provided in the article, and that the summary coding table is detailed and thorough, so a reader can understand how the researchers reached their conclusions. Nevertheless, I would recommend additional information in the Data Availability statement to explicitly address the issue of access to data. | Thank you for this feedback. We have provided updated information for the Data Availability statement to address this. |

The following changes to our reference list have been made as the publication status of some of the articles cited has changed and we have removed Supplementary Item 6 Unpublished citations (S6_File):

- S6.1 has been added to the reference list as:
  - Wang, HI., Wright, B., Tindall, L., Cooper, C., Biggs, K., Lee, E., et al. Cost and effectiveness of one session treatment (OST) for children and young people with specific phobias compared to multi-session cognitive behavioural therapy (CBT): results from a randomised controlled trial. BMC Psychiatry. 2022;22(1):547.
- S6.2 has been added to the reference list as:
  - Wright B, Tindall L, Scott AJ, Lee E, Cooper C, Biggs K, et al. One session treatment (OST) is equivalent to multi-session cognitive behavioral therapy (CBT) in children with specific phobias (ASPECT): results from a national non-inferiority randomized controlled trial. J Child Psychol Psychiatry. Epub ahead of print 2022 Aug 1. Available from: <https://pubmed.ncbi.nlm.nih.gov/35915056/>
- An erratum has been added to the reference Hofman et al (2012):
  - Hofmann SG, Asnaani A, Vonk IJJ, Sawyer AT, Fang A. The efficacy of cognitive behavioral therapy: A review of meta-analyses. Cognit Ther Res. 2012;36(5):427–40. [Erratum in: Cognit Ther Res 2014; 38: 368]
